# The First-Principles Study of External Strain Tuning the Electronic and Optical Properties of the 2D MoTe_2_/PtS_2_ van der Waals Heterostructure

**DOI:** 10.3389/fchem.2022.934048

**Published:** 2022-07-25

**Authors:** Li Zhang, Kai Ren, Haiyan Cheng, Zhen Cui, Jianping Li

**Affiliations:** ^1^ Department of Application & Engineering, Zhejiang Institute of Economics and Trade, Hangzhou, China; ^2^ School of Mechanical and Electronic Engineering, Nanjing Forestry University, Nanjing, China; ^3^ School of Mechanical Engineering, Wanjiang University of Technology, Maanshan, China; ^4^ School of Foreign Languages, Zhejiang University of Finance & Economics Dongfang College, Zhejiang, China; ^5^ School of Automation and Information Engineering, Xi’an University of Technology, Xi’an, China; ^6^ School of Automotive & Transportation Engineering, Shenzhen Polytechnic, Shenzhen, China

**Keywords:** vdW heterostructures, first-principles method, MoTe_2_/PtS_2_, strain, solar-to-hydrogen efficiency

## Abstract

Two-dimensional van der Waals (vdW) heterostructures reveal novel properties due to their unique interface, which have attracted extensive focus. In this work, the first-principles methods are explored to investigate the electronic and the optical abilities of the heterostructure constructed by monolayered MoTe_2_ and PtS_2_. Then, the external biaxial strain is employed on the MoTe_2_/PtS_2_ heterostructure, which can persist in the intrinsic type-II band structure and decrease the bandgap. In particular, the MoTe_2_/PtS_2_ vdW heterostructure exhibits a suitable band edge energy for the redox reaction for water splitting at pH 0, while it is also desirable for that at pH 7 under decent compressive stress. More importantly, the MoTe_2_/PtS_2_ vdW heterostructure shows a classy solar-to-hydrogen efficiency, and the light absorption properties can further be enhanced by the strain. Our results showed an effective theoretical strategy to tune the electronic and optical performances of the 2D heterostructure, which can be used in energy conversion such as the automotive battery system.

## Introduction

Graphene shows unique electronic and thermal performances after being prepared as a two-dimensional (2D) material (Geim and Novoselov, 2007), which has also attracted other layered materials (Cui et al., 2021a; Cui et al., 2021b; Ren et al., 2022a; Wang et al., 2022a). However, its zero bandgap restricts the applications as electronic switch and other devices. Therefore, 2D semiconducting materials include transition metal dichalcogenides (TMDs) (Shen et al., 2022), phosphorene, and MXenes. MoS_2_ possesses excellent electronic and photoelectric properties similar to or even more advantageous than graphene in some aspects (Butler et al., 2013; Zhang et al., 2018). After the successful synthesis of graphene and MoS_2_, more and more 2D materials have been found and synthesized. Its direct bandgap is about ∼1.8 eV (Wickramaratne et al., 2014), which can be widely used in transistors, optoelectronics, and photocatalysts (Radisavljevic et al., 2011; Qiu et al., 2013; Ma et al., 2020). Black phosphorene has intrinsic direct bandgap and high carrier mobility (Li et al., 2014). Due to the anisotropic structure, black phosphorene shows remarkable anisotropic electronic, mechanical, and thermal properties. All these excellent properties endow its application in high-performance photovoltaic (Liu et al., 2017), spin-filter devices (You et al., 2016), thermal rectifiers (Ren et al., 2020a), field-effect transistors (Hong et al., 2014), etc.

To expand the family of 2D materials, tremendous investigations have been conducted to predict the structure and properties of these layered materials (Sun et al., 2020; Sun and Schwingenschlögl, 2020; Sun et al., 2021; Sun and Schwingenschlögl, 2021). In addition, the formation of the van der Waals (vdW) heterostructure by different 2D materials is also a popular strategy to extend the applications of the 2D materials (Wang et al., 2022b). The vdW interactions in the heterostructure result in novel interfacial performances, which can improve the electronic (Ren et al., 2021a), optical (Ren et al., 2019a), and catalytic (Wang et al., 2018a; Wang et al., 2020a; Wang et al., 2020b) characteristics. Furthermore, such excellent properties of the heterostructure can even be tuned by the electric field (Sun et al., 2017a), strain (Ren et al., 2019b; Wang et al., 2020c; Wang et al., 2020d), stacking (Ren et al., 2022b), doping (Ren et al., 2022c),and defect (Sun et al., 2017b). When applied, the external strain is an effective tactic; for example, the band structure of the MXene/blue phosphorene vdW heterostructure can result in the transformation from type-I to type-II by the strain (Guo et al., 2017). The external strain also possesses a significant influence on the layer distance, which further decides the interfacial performances (Guo et al., 2020). Under the strain, the evolution of Schottky barriers of the GaN/graphene heterostructure can be converted from the n-Schottky to Ohmic type (Deng and Wang, 2019). Recently, the TMD materials of MoTe_2_ and PtS_2_ monolayers have been prepared experimentally (Qu et al., 2017; Zhao et al., 2019). The MoTe_2_ and PtS_2_ monolayers present the novel electronic (Qu et al., 2017; Sajjad et al., 2018) and thermoelectric (Shi et al., 2017) properties, which have been widely studied. The 2D MoTe_2_ can be obtained from mechanically exfoliated bulk crystals (Chang et al., 2016), which have potential usages in electronics, such as inverters and amplifiers, and in logic and digital circuits. Moreover, the electronic property of MoTe_2_ is sensitive to atomic doping (Kanoun, 2018), suggesting tunable electronic and optical performances. The PtS_2_ monolayer also presents tunable properties by the strain (Liu et al., 2018) and electric field (Nguyen et al., 2019). In addition, the PtS_2_ monolayer is reported to be formed as a vdW heterostructure such as PtS_2_/arsenene (Ren et al., 2020b), PtS_2_/InSe (Nguyen et al., 2019), and HfS_2_/PtS_2_ (Colibaba et al., 2019). Furthermore, the MoTe_2_ and PtS_2_ monolayers share a honeycomb hexagonal structure with a small lattice mismatch, explaining the advantage to be formed as a heterostructure. Therefore, the MoTe_2_ and PtS_2_ monolayers have been decided to be used for constructing the heterostructure in this work. The first-principles calculations are developed to investigate the band structure of the MoTe_2_/PtS_2_ (MP) heterostructure. Importantly, the tunable electronic, charge density, potential, light absorption ability, and the solar-to-hydrogen efficiency (STH) by the external biaxial strain are addressed.

## Computational Methods

In this investigation, the Vienna *ab initio* simulation software package (VASP) was used to find the first-principles simulations by the density functional theory (DFT) (Kresse and Furthmüller, 1996a; Kresse and Furthmüller, 1996b). In the generalized gradient approximation (GGA), the projector augmented wave (PAW) potentials were used with the Perdew–Burke–Ernzerhof (PBE) functional to describe the core electrons and the exchange–correlation functional (Perdew et al., 1996; Kresse and Joubert, 1999). The cut-off energy was used by 550 eV, and the Monkhorst–Pack *k*-point was 15 × 15 × 1 in the calculations. Furthermore, the Heyd–Scuseria–Ernzerhof hybrid method was adopted to calculate the electronic and optical properties (Heyd et al., 2005). The weak dispersion forces were described by the DFT-D3 method proposed by Grimme et al. (2010). Due to the ignorable effect of the spin–orbit coupling (SOC) on the electronic properties of the studied system, shown in Supplementary Figure S1, the SOC is not employed in the calculations. The vacuum thickness was set as 25 Å to prohibit the interaction adjacent layers. Besides, the convergence criterion of the force in the simulations was 0.01 eV Å^−1^, while the energy was controlled in 0.01 meV.

## Results and Discussion

**FIGURE 1:**
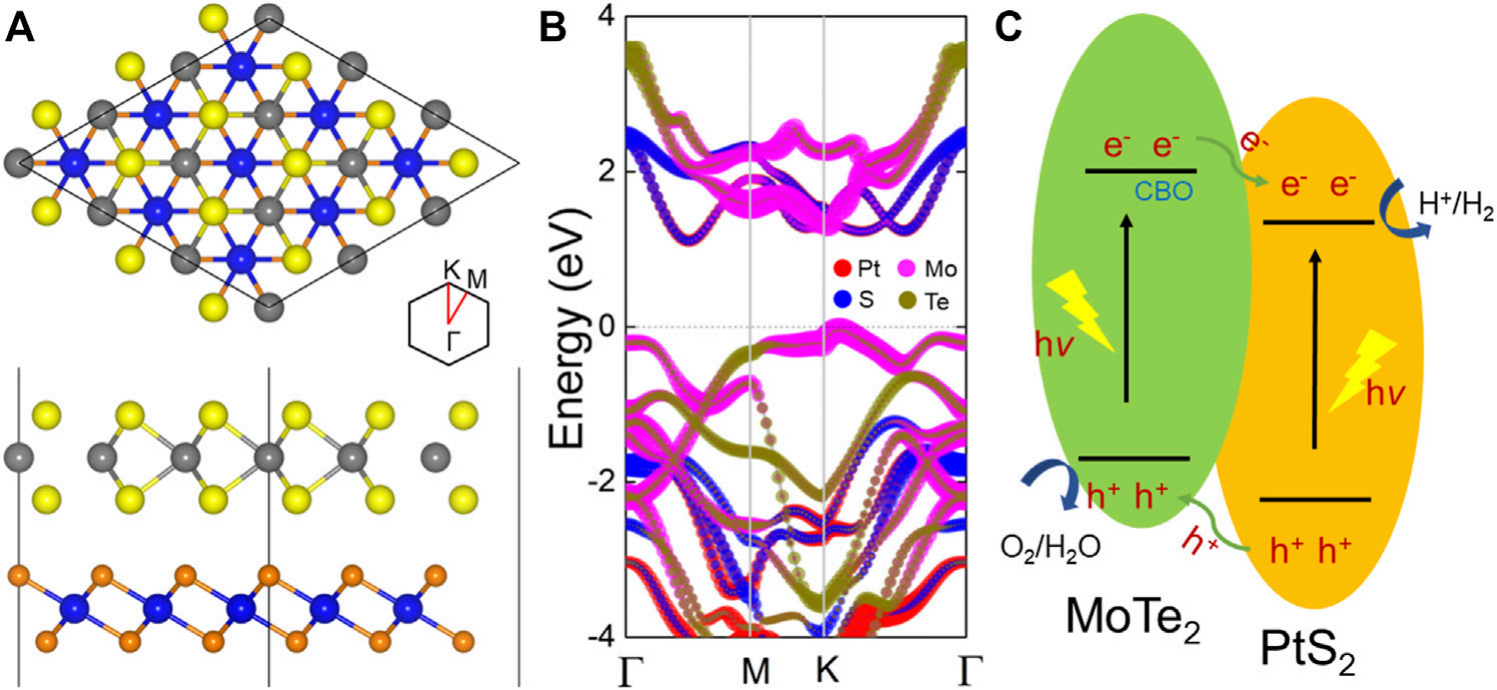
**(A)** Geometric and **(B)** band structure of the MP heterostructure with the lowest binding energy. **(C)** Photogenerated charge migration path in the MP vdW heterostructure. The yellow, gray, orange, and blue spheres represent the Te, Mo, S, and Pt atoms, respectively. The Fermi level is decided as 0 shown by the gray dashed line.

The lattice parameters of the MoTe_2_ and PtS_2_ monolayers are optimized as 3.564 and 3.529 Å, respectively, showing a low lattice mismatch of about 0.1%, which are suitable to be constructed as a heterostructure. Supplementary Figure S2 shows the band structures of the pristine MoTe_2_ and PtS_2_ monolayers calculated using the HSE06 functional with the indirect and direct bandgaps of 1.22 and 2.60 eV, respectively, demonstrating an agreement with the previous reports (Shao et al., 2022). Then, the MP heterostructure is constructed by considering six different highly symmetrical structures, as shown in Supplementary Figure S3. By calculating the binding energy, the most stable stacking configuration is decided, as shown in Figure 1A, that the Mo atoms are located on top of the upper S atoms, while the Te atoms are set on top of the lower S atoms. The MoTe_2_/PtS_2_ heterostructure is built by vdW forces because of the weak binding energy of about −28.10 meV Å^−2^ (Ren et al., 2022b), which is lower than that in graphites (about −18 meV Å^−2^) (Chen et al., 2013). The projected band structure of the MP vdW heterostructure is obtained in Figure 1B, suggesting an indirect bandgap of about 1.26 eV. The CBM and the VBM of the MP vdW heterostructure result from the PtS_2_ and MoTe_2_ monolayers, respectively, showing a type-II band alignment, which can separate the photogenerated electrons and holes using as a photocatalyst for water splitting (Ren et al., 2021b). In detail, when the MP vdW heterostructure obtains the energy from the light, the photogenerated electrons will move to the conduction band of the MoTe_2_ and PtS_2_ monolayers, as shown in Figure 1C, resulting in photogenerated holes staying at the valence band. Then, the conduction band offset (CBO) can promote the photogenerated electrons from MoTe_2_ to PtS_2_ at the conduction band, while the photogenerated holes will be transferred from PtS_2_ to MoTe_2_ at the valence band. Thus, the photogenerated charges in the MP vdW heterostructure are prevented from recombination.

**FIGURE 2:**
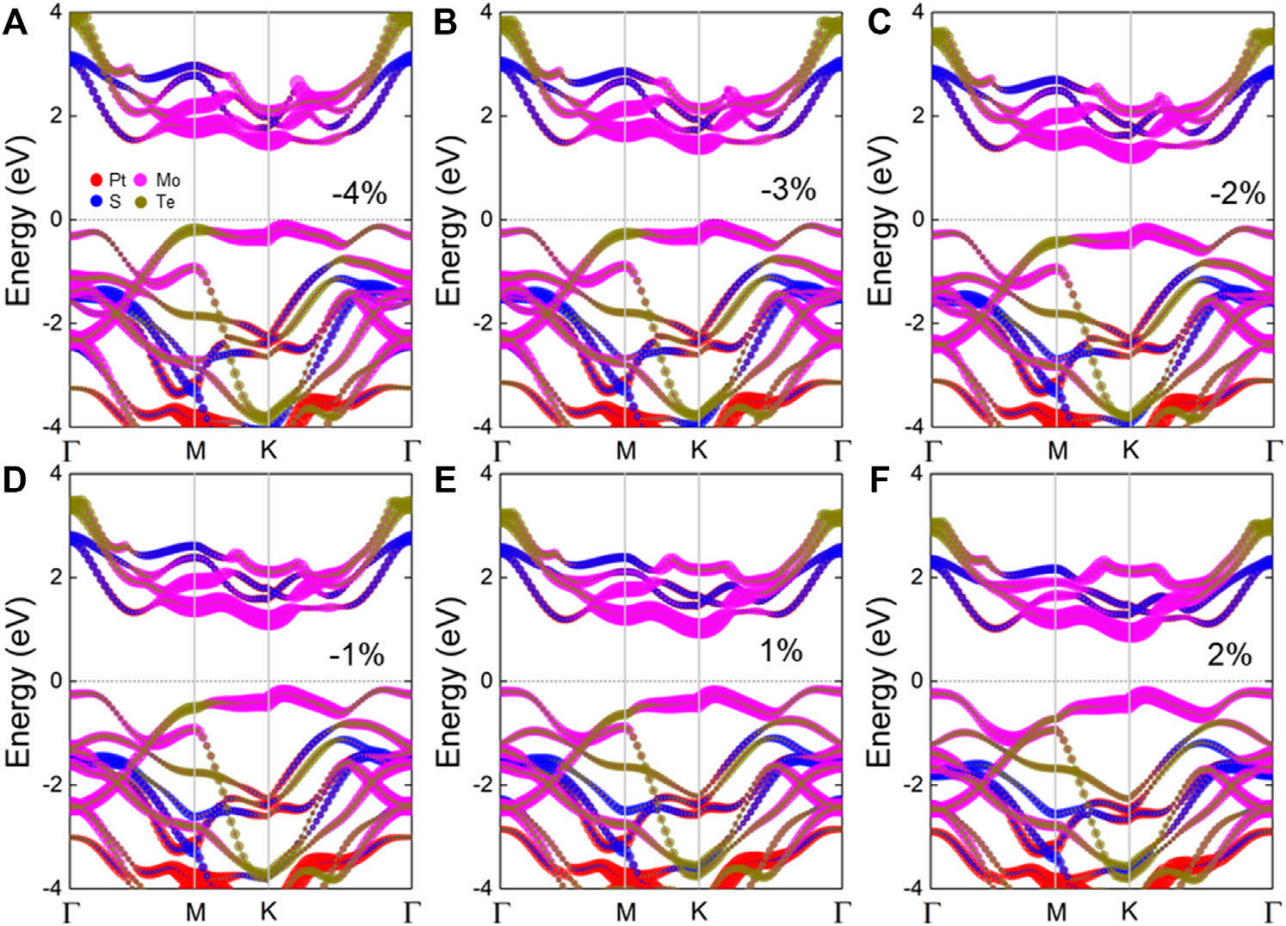
Band structures of the MP vdW heterostructure under the external strains of **(A)** –4%, **(B)** –3%, **(C)** –2%, **(D)** –1%, **(E)** 1%, and **(F)** 2%.

**FIGURE 3:**
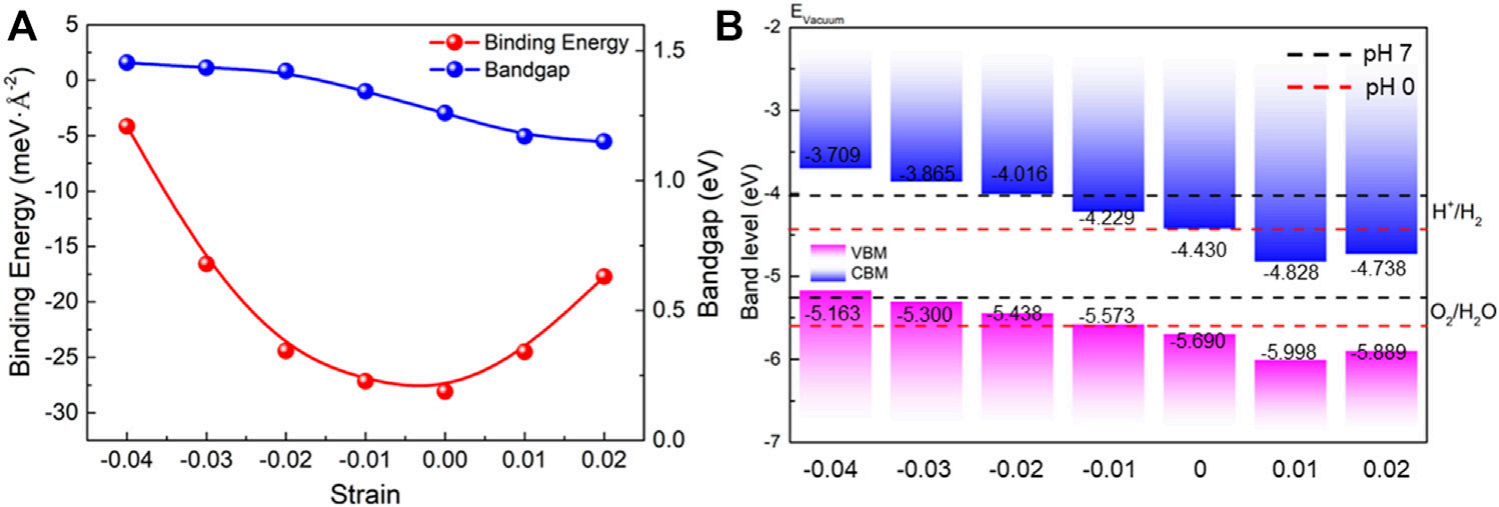
**(A)** Band alignment of the MP vdW heterostructure by the external strain comparing the energy potential of the redox reaction at pH values 0 and 7. **(B)** Binding energy and the bandgap difference of the MP vdW heterostructure tuned by the external strain.

Next, the external biaxial strain is applied in the MP vdW heterostructure to explore its effect on the electronic structure. In Figure 2, the projected band structure of the MP vdW heterostructure under the external biaxial strain from –4 to 2% is obtained, where negative and positive values represent pressure and tension, respectively. One can see that the type-II band structure is retained in the MP vdW heterostructure with that strain, which still can separate the photogenerated electrons and holes, while the bandgap decreased from 1.454 to 1.150 eV by the external biaxial strain from –4 to 2%, as shown in Figure 3A. In addition, the binding energy (*E*_b_) is also investigated, which is decided by

$$E_{b}=E_{H}-E_{M}-E_{P},$$ (1)

where *E*_H_, *E*_M_, and *E*_P_ represent the total energy of the MP vdW heterostructure, pristine MoTe_2_, and PtS_2_, respectively. The calculated binding energy change of the MP vdW heterostructure applied by different external biaxial strains is demonstrated by Figure 3B, which shows the stability of the MP vdW heterostructure, and the lowest binding energy of the MP vdW heterostructure is the unstressed state.

The band edge positions of the MP vdW heterostructure under different strains are also calculated by the HSE06 functional, demonstrated in Figure 3B. The potential energy values of the oxidation and reduction reactions for water splitting are –5.67 eV and –4.44 eV, respectively, at pH 0. The energy of the redox potential can be decided by the pH level with: *E* = −4.44 eV + pH × 0.059 eV for the reduction reaction, while the potential of the oxidation is obtained by *E* = −5.67 eV + pH × 0.059 eV. Thus, the calculated potentials of the oxidation and reduction reactions at pH 0 are −5.26 eV and −4.03 eV, respectively, at pH 7. As a decent photocatalyst, the band edge positions of the CBM (or VBM) of the heterostructure should be higher (or lower) than the potential of the reduction (or oxidation) for water splitting (Ren et al., 2021c). In Figure 3B, one can see that the MP vdW heterostructure possesses suitable band edge positions to promote the redox reaction at pH 7 for water splitting by the external biaxial strains of –3% and –2%, while the MP vdW heterostructure can be used as a promising photocatalyst for water splitting at pH 0 without the external strain.

**FIGURE 4:**
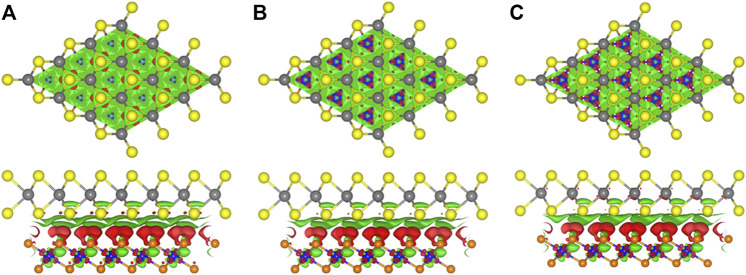
Isosurface of the charge density difference of the MP vdW heterostructure under the strains of **(A)** –4%, **(B)** –2%, and **(C)** 2%; red and green marks demonstrate the losing and gaining of electrons, respectively. The isosurface parameter is 0.001 |e|.

The charge density difference (Δ*ρ*) of the MP vdW heterostructure tuned by the strain is also investigated, which is calculated as follows:

Δρ=ρH−ρM−ρP, (2)

where *ρ*_H_, *ρ*_M,_ and *ρ*_P_ are used as the charge densities of the MP vdW heterostructure, pristine MoTe_2_, and PtS_2_, respectively. Under these strains, the PtS_2_ layer still gains the electrons from the MoTe_2_ layer, and the charge density difference between the interface of the MP vdW heterostructure under –4%, –2%, and 2% is demonstrated in Figures 4A–C, respectively. In addition, the quantitative analysis of the charge transfer in the MP vdW heterostructure is explored by the Bader charge method (Sanville et al., 2007). The calculated charge transfers between the interface of the MP vdW heterostructure under –4%, –2%, and 2% are 0.0463 |e|, 0.0475 |e|, and 0.052 |e|, respectively.

**FIGURE 5:**
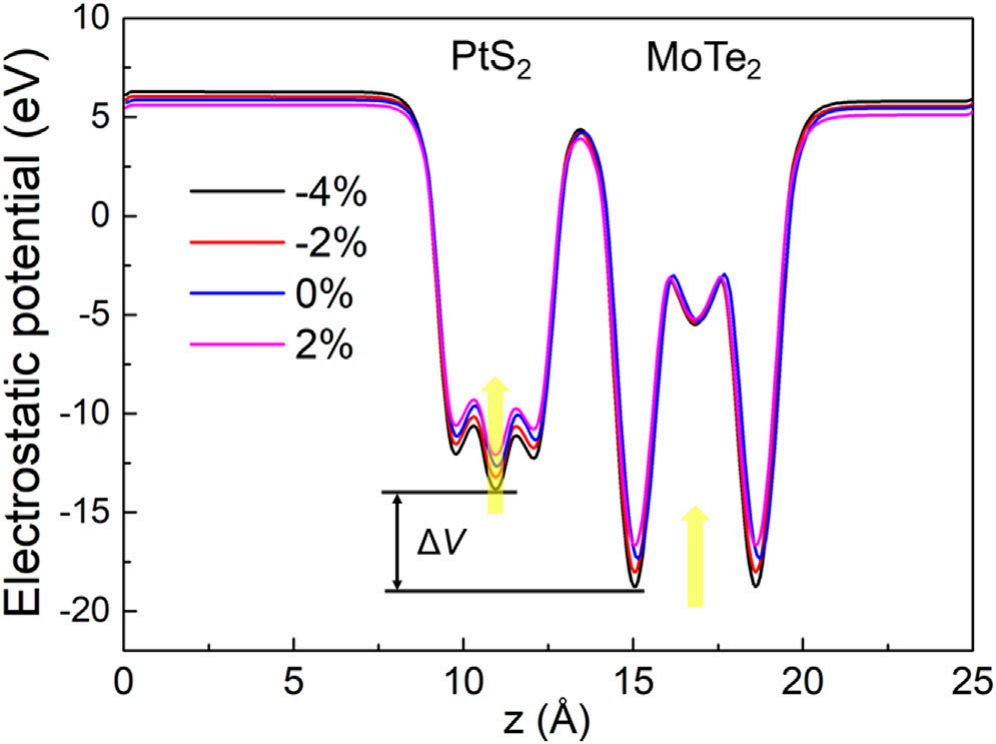
Potential drop of the MP vdW heterostructure across the interface by different external strains.

The charge density difference between the interface of the MoTe_2_ and PtS_2_ monolayers can induce a potential drop. The potential energy of the MoTe_2_ and PtS_2_ in the heterostructure by the different strains is investigated in Figure 5, showing that the strain can increase the potential energy of the MoTe_2_ and PtS_2_ from pressure to tension. The potential drop across the interface of the MP vdW heterostructure is obtained as 4.962, 4.720, 4.672, and 4.500 eV by the external biaxial strains of –4%, –2%, –0%, and 2%, respectively, which demonstrates the decreased charge density difference. It is worth emphasizing that such a potential drop in the MP vdW heterostructure can also provide a critical boost for the separation of the photogenerated electrons and holes.

**FIGURE 6:**
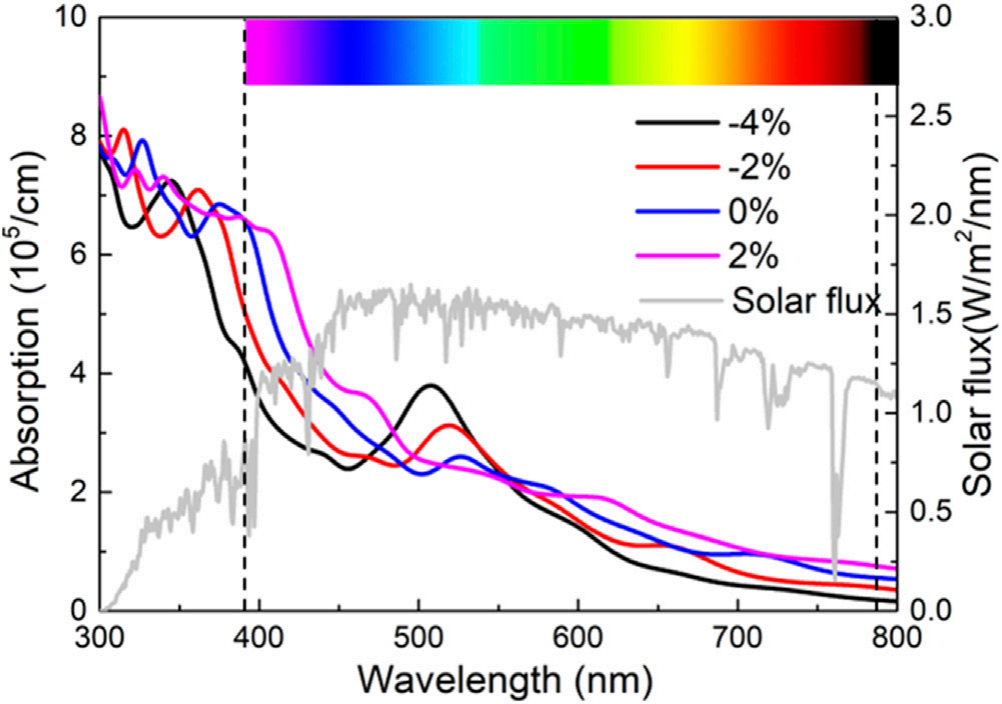
Calculated optical absorption of the MP vdW heterostructure under the external strain.

We also studied the light absorption coefficient (*α*) of the MP vdW heterostructure by external strain using the HSE06 method, which is calculated as follows:

$$\alpha \left(\omega \right)=\frac{\sqrt{2}\omega }{c}{\left\{{\left[\varepsilon_{1}^{2}\left(\omega \right)+\varepsilon_{2}^{2}\left(\omega \right)\right]}^{1/2}-\varepsilon_{1}\left(\omega \right)\right\}}^{1/2},$$ (3)

where *ω* is the angular frequency, and *c* is the speed of light. The real and imaginary parts are represented by *ε*_1_(*ω*) and *ε*_2_(*ω*), respectively. Moreover, *ε*_1_(*ω*) and *ε*_2_(*ω*) can be obtained as follows:

$$\varepsilon_{2}\left(q\to O_{\hat{u}},\hbar \omega \right)=\frac{2e^{2}\pi }{\Omega \varepsilon_{0}}{\sum_{k,v,c}\left|\langle \Psi_{k}^{c}\right|\hat{u}\cdot r\left|\Psi_{k}^{v}\rangle \right|}^{2}\times \delta \left(E_{k}^{c}-E_{k}^{v}-E\right),$$ (4)

$$\varepsilon \left(\omega \right)=\varepsilon_{1}\left(\omega \right)+i\varepsilon_{2}\left(\omega \right),$$ (5)

where $\Psi_{k}$ , $E_{k}$ , and $\hat{u}$ are the wave function, energy, and unit vector of the electric field of the incident light, respectively. The superscripts (*v* and *c*) in $\Psi_{k}$ and $E_{k}$ are labeled as the conduction bands and valence bands, respectively. The calculated light absorption performance of the strained MP vdW heterostructure is explained by Figure 6 marked by a visible spectrum (Wang et al., 2018b). Evidently, applying the external compressive stress can improve the light absorption capacity at the absorption wavelength ranging from 480 to 550 nm. In detail, the light absorption peaks of the MP vdW heterostructure are obtained as 3.79 × 10^5^ cm^−1^, 3.13 × 10^5^ cm^−1^, and 2.60 × 10^5^ cm^−1^ locating the wavelength at 509 nm, 519, and 527 nm, respectively, by the strains of –0.04, –0.02, and 0, while the light absorption performance of the MP vdW heterostructure can be enhanced by tensile stress when the absorption wavelength exceeds 550 nm. Furthermore, these obtained absorption performances are also higher than those of other 2D heterostructures, such as CdO/HfS_2_ (3.51 × 10^5^ cm^−1^) (Zhang et al., 2022), arsenene/PtSe_2_ (2.23 × 10^5^ cm^−1^) (Zheng et al., 2021), and MoSSe/GaN (2.74 × 10^5^ cm^−1^) (Ren et al., 2020c).

Furthermore, the solar-to-hydrogen efficiency of the MP vdW heterostructure is calculated by *η*_STH_ = *η*_abs_ × *η*_cu_ (Fu et al., 2018), where *η*_abs_ is the efficiency of light absorption, and *η*_cu_ demonstrates carrier utilization. As for the light absorption, it can be decided by

$$\eta_{abs}=\frac{\int_{E_{g}}^{\infty }P\left(\hbar \omega \right)d\left(\hbar \omega \right)}{\int_{0}^{\infty }P\left(\hbar \omega \right)d\left(\hbar \omega \right)},$$ (6)

where *E*_g_ means the bandgap of the studied material. ℏω is used to explain the photon energy, while the AM1.5G solar energy flux is *P*(*hω*). The carrier utilization is obtained by

$$\eta_{\mathrm{cu}}=\frac{\Delta G\int_{E}^{\infty }\frac{P\left(\hbar \omega \right)}{\hbar \omega }d\left(\hbar \omega \right)}{\int_{E_{g}}^{\infty }P\left(\hbar \omega \right)d\left(\hbar \omega \right)}.$$ (7)

To describe the potential difference in water splitting, Δ*G* is used by 1.23 eV here. Importantly, the photon energy of *E* is calculated by

$$E=\{\begin{array}{r} E_{g},\left(\chi \left(H_{2}\right)\geq 0.2,\chi \left(O_{2}\right)\geq 0.6\right),\\ E_{g}+0.2-\chi \left(H_{2}\right),\left(\chi \left(H_{2}\right)<0.2,\chi \left(O_{2}\right)\geq 0.6\right),\\ E_{g}+0.6-\chi \left(O_{2}\right),\left(\chi \left(H_{2}\right)\geq 0.2,\chi \left(O_{2}\right)<0.6\right),\\ E_{g}+0.8-\chi \left(H_{2}\right)-\chi \left(O_{2}\right),\left(\chi \left(H_{2}\right)<0.2,\chi \left(O_{2}\right)<0.6\right). \end{array}$$ (8)

The overpotential for the reduction and oxidation reactions for water splitting is explained by *χ*(H_2_) and *χ*(O_2_), respectively. In addition, previous experimental investigations provide the necessary overpotentials for the reduction and oxidation reactions as 0.2 and 0.6 eV (Fu et al., 2018), respectively. The efficiencies of light absorption of the MP vdW heterostructure under the strains of –0.03, –0.02, and 0 are calculated by 89.44, 89.80, and 93.26%, respectively. Furthermore, the carrier utilization of the MP vdW heterostructure is 33.66, 33.53, and 32.28%, respectively, by the strains of –0.03, –0.02, and 0. Thus, the solar-to-hydrogen efficiencies are obtained as 30.10, 30.11, and 30.10%, respectively, at pH values 7, 7, and 0, as shown in Table. 1, which is higher than other 2D heterostructures such as CdO/arsenene (about 11.67%) (Ren et al., 2021b) and GaS/arsenene (about 25.46%) (Li et al., 2021).

**TABLE 1:** Energy conversion efficiency of light absorption, carrier utilization, and the solar-to-hydrogen efficiency of the MP vdW heterostructure under the external strain.

Strain	*η*_abs_ (%)	*η*_cu_ (%)	*η*_STH_ (%)
–0.03	89.44	33.66	30.10
–0.02	89.80	30.11	30.11
0	93.26	30.10	30.10

## Conclusion

In this work, the first-principles method is employed to investigate the electronic and optical performances of the MP vdW heterostructure. The external strain is also applied on the MP vdW heterostructure, and the results show that the MP vdW heterostructure maintains the type-II band alignment and decreased bandgap. In addition, the external compressive stress can tune the MP vdW heterostructure as a potential for water splitting at pH 7 because of the decent band edge positions. The strain also has a significant influence on the interfacial properties of the MP vdW heterostructure. Furthermore, the MP vdW heterostructure possesses excellent light absorption capacity and light conversion efficiency, which can also be enhanced by the strain. The results show that the MP vdW heterostructure possesses potential energy conversion used in the automotive battery system.

## Data Availability

The raw data supporting the conclusions of this article will be made available by the authors, without undue reservation.

## References

[B1] Butler S. Z. Hollen S. M. Cao L. Cui Y. Gupta J. A. Gutiérrez H. R. (2013). Progress, Challenges, and Opportunities in Two-Dimensional Materials beyond Graphene. ACS Nano 7, 2898–2926. 10.1021/nn400280c 23464873

[B2] Chang Y.-M. Lin C.-Y. Lin Y.-F. Tsukagoshi K. (2016). Two-Dimensional MoTe_2_ Materials: From Synthesis, Identification, and Charge Transport to Electronics Applications. Jpn. J. Appl. Phys. 55, 1102A1101. 10.7567/jjap.55.1102a1

[B3] Chen X. Tian F. Persson C. Duan W. Chen N.-x. (2013). Interlayer Interactions in Graphites. Sci. Rep. 3, 3046. 10.1038/srep03046 24192753 PMC3818654

[B4] Colibaba S. A. Körbel S. Motta C. El-Mellouhi F. Sanvito S. (2019). Interlayer dielectric function of a type-II van der Waals semiconductor: The HfS2/PtS2 heterobilayer. Phys. Rev. Mater. 3, 124002. 10.1103/physrevmaterials.3.124002

[B5] Cui Z. Luo Y. Yu J. Xu Y. (2021). Tuning the Electronic Properties of MoSi2N4 by Molecular Doping: A First Principles Investigation. Phys. E Low-dimensional Syst. Nanostructures 134, 114873. 10.1016/j.physe.2021.114873

[B6] Cui Z. Wang M. Lyu N. Zhang S. Ding Y. Bai K. (2021). Electronic, Magnetism and Optical Properties of Transition Metals Adsorbed Puckered Arsenene. Superlattices Microstruct. 152, 106852. 10.1016/j.spmi.2021.106852

[B7] Deng Z. Wang X. (2019). Strain Engineering on the Electronic States of Two-Dimensional GaN/graphene Heterostructure. RSC Adv. 9, 26024–26029. 10.1039/c9ra03175h 35531004 PMC9070312

[B8] Fu C.-F. Sun J. Luo Q. Li X. Hu W. Yang J. (2018). Intrinsic Electric Fields in Two-Dimensional Materials Boost the Solar-To-Hydrogen Efficiency for Photocatalytic Water Splitting. Nano Lett. 18, 6312–6317. 10.1021/acs.nanolett.8b02561 30238753

[B9] Geim A. K. Novoselov K. S. (2007). The Rise of Graphene. Nat. Mater 6, 183–191. 10.1038/nmat1849 17330084

[B10] Grimme S. Antony J. Ehrlich S. Krieg H. (2010). A Consistent and Accurate Ab Initio Parametrization of Density Functional Dispersion Correction (DFT-D) for the 94 Elements H-Pu. J. Chem. Phys. 132, 154104. 10.1063/1.3382344 20423165

[B11] Guo W. Ge X. Sun S. Xie Y. Ye X. (2020). The strain effect on the electronic properties of the MoSSe/WSSe van der Waals heterostructure: a first-principles study. Phys. Chem. Chem. Phys. 22, 4946–4956. 10.1039/d0cp00403k 32073069

[B12] Guo Z. Miao N. Zhou J. Sa B. Sun Z. (2017). Strain-mediated type-I/type-II transition in MXene/Blue phosphorene van der Waals heterostructures for flexible optical/electronic devices. J. Mat. Chem. C 5, 978–984. 10.1039/c6tc04349f

[B13] Heyd J. Peralta J. E. Scuseria G. E. Martin R. L. (2005). Energy Band Gaps and Lattice Parameters Evaluated with the Heyd-Scuseria-Ernzerhof Screened Hybrid Functional. J. Chem. Phys. 123, 174101. 10.1063/1.2085170 16375511

[B14] Hong T. Chamlagain B. Lin W. Chuang H.-J. Pan M. Zhou Z. (2014). Polarized Photocurrent Response in Black Phosphorus Field-Effect Transistors. Nanoscale 6, 8978–8983. 10.1039/c4nr02164a 24967826

[B15] Kanoun M. B. (2018). Tuning Magnetic Properties of Two-Dimensional MoTe2 Monolayer by Doping 3d Transition Metals: Insights from First Principles Calculations. J. Alloys Compd. 748, 938–942. 10.1016/j.jallcom.2018.03.132

[B16] Kresse G. Furthmüller J. (1996). Efficiency of Ab-Initio Total Energy Calculations for Metals and Semiconductors Using a Plane-Wave Basis Set. Comput. Mater. Sci. 6, 15–50. 10.1016/0927-0256(96)00008-0

[B17] Kresse G. Furthmüller J. (1996). Efficient Iterative Schemes Forab Initiototal-Energy Calculations Using a Plane-Wave Basis Set. Phys. Rev. B 54, 11169–11186. 10.1103/physrevb.54.11169 9984901

[B18] Kresse G. Joubert D. (1999). From Ultrasoft Pseudopotentials to the Projector Augmented-Wave Method. Phys. Rev. B 59, 1758–1775. 10.1103/physrevb.59.1758

[B19] Li J. Huang Z. Ke W. Yu J. Ren K. Dong Z. (2021). High solar-to-hydrogen efficiency in Arsenene/GaX (X = S, Se) van der Waals heterostructure for photocatalytic water splitting. J. Alloys Compd. 866, 158774. 10.1016/j.jallcom.2021.158774

[B20] Li L. Yu Y. Ye G. J. Ge Q. Ou X. Wu H. (2014). Black Phosphorus Field-Effect Transistors. Nat. Nanotech 9, 372–377. 10.1038/nnano.2014.35 24584274

[B21] Liu G. Gan Y. Quhe R. Lu P. (2018). Strain Dependent Electronic and Optical Properties of PtS2 Monolayer. Chem. Phys. Lett. 709, 65–70. 10.1016/j.cplett.2018.08.029

[B22] Liu Y. Cai Y. Zhang G. Zhang Y.-W. Ang K.-W. (2017). Al-Doped Black Phosphorus P-N Homojunction Diode for High Performance Photovoltaic. Adv. Funct. Mat. 27, 1604638. 10.1002/adfm.201604638

[B23] Ma L. Xu Y. Zheng J. Dai X. (2020). Ecodesign Method of Intelligent Boom Sprayer Based on Preferable Brownfield Process. J. Clean. Prod. 268, 122206. 10.1016/j.jclepro.2020.122206

[B24] Nguyen C. V. Bui H. D. Nguyen T. D. Pham K. D. (2019). Controlling electronic properties of PtS2/InSe van der Waals heterostructure via external electric field and vertical strain. Chem. Phys. Lett. 724, 1–7. 10.1016/j.cplett.2019.03.048

[B25] Perdew J. P. Burke K. Ernzerhof M. (1996). Generalized Gradient Approximation Made Simple. Phys. Rev. Lett. 77, 3865–3868. 10.1103/physrevlett.77.3865 10062328

[B26] Qiu D. Y. da Jornada F. H. Louie S. G. (2013). Optical Spectrum ofMoS2: Many-Body Effects and Diversity of Exciton States. Phys. Rev. Lett. 111, 216805. 10.1103/physrevlett.111.216805 24313514

[B27] Qu D. Liu X. Huang M. Lee C. Ahmed F. Kim H. (2017). Carrier-Type Modulation and Mobility Improvement of Thin MoTe2. Adv. Mat. 29, 1606433. 10.1002/adma.201606433 28845903

[B28] Radisavljevic B. Radenovic A. Brivio J. Giacometti V. Kis A. (2011). Single-layer MoS2 Transistors. Nat. Nanotech 6, 147–150. 10.1038/nnano.2010.279 21278752

[B29] Ren K. Liu X. Chen S. Cheng Y. Tang W. Zhang G. (2020). Remarkable Reduction of Interfacial Thermal Resistance in Nanophononic Heterostructures. Adv. Funct. Mat. 30, 2004003. 10.1002/adfm.202004003

[B30] Ren K. Luo Y. Wang S. Chou J.-P. Yu J. Tang W. (2019). A van der Waals Heterostructure Based on Graphene-like Gallium Nitride and Boron Selenide: A High-Efficiency Photocatalyst for Water Splitting. ACS Omega 4, 21689–21697. 10.1021/acsomega.9b02143 31891047 PMC6933577

[B31] Ren K. Qin H. Liu H. Chen Y. Liu X. Zhang G. (2022). Manipulating Interfacial Thermal Conduction of 2D Janus Heterostructure via a Thermo‐Mechanical Coupling. Adv. Funct. Mater. 32, 2110846. 10.1002/adfm.202110846

[B32] Ren K. Shu H. Huo W. Cui Z. Xu Y. (2022c). Tuning Electronic, Magnetic and Catalytic Behaviors of Biphenylene Network by Atomic Doping. Nanotechnology 33, 345701. 10.1088/1361-6528/ac6f64 35561655

[B33] Ren K. Shu H. Huo W. Cui Z. Yu J. Xu Y. (2021). Mechanical, Electronic and Optical Properties of a Novel B2P6 Monolayer: Ultrahigh Carrier Mobility and Strong Optical Absorption. Phys. Chem. Chem. Phys. 23, 24915–24921. 10.1039/d1cp03838a 34726209

[B34] Ren K. Sun M. Luo Y. Wang S. Yu J. Tang W. (2019). First-principle Study of Electronic and Optical Properties of Two-Dimensional Materials-Based Heterostructures Based on Transition Metal Dichalcogenides and Boron Phosphide. Appl. Surf. Sci. 476, 70–75. 10.1016/j.apsusc.2019.01.005

[B35] Ren K. Tang W. Sun M. Cai Y. Cheng Y. Zhang G. (2020). A direct Z-scheme PtS2/arsenene van der Waals heterostructure with high photocatalytic water splitting efficiency. Nanoscale 12, 17281–17289. 10.1039/d0nr02286a 32633304

[B36] Ren K. Wang S. Luo Y. Chou J.-P. Yu J. Tang W. (2020). High-efficiency photocatalyst for water splitting: a Janus MoSSe/XN (X = Ga, Al) van der Waals heterostructure. J. Phys. D. Appl. Phys. 53, 185504. 10.1088/1361-6463/ab71ad

[B37] Ren K. Zheng R. Xu P. Cheng D. Huo W. Yu J. (2021). Electronic and Optical Properties of Atomic-Scale Heterostructure Based on MXene and MN (M = Al, Ga): A DFT Investigation. Nanomaterials 11, 2236. 10.3390/nano11092236 34578552 PMC8467826

[B38] Ren K. Zheng R. Yu J. Sun Q. Li J. (2021). Band Bending Mechanism in CdO/Arsenene Heterostructure: A Potential Direct Z-Scheme Photocatalyst. Front. Chem. 9, 788813. 10.3389/fchem.2021.788813 34869235 PMC8641692

[B39] Ren K. Zhu Z. Wang K. Huo W. Cui Z. (2022). Stacking-Mediated Type-I/Type-II Transition in Two-Dimensional MoTe2/PtS2 Heterostructure: A First-Principles Simulation. Crystals 12, 425. 10.3390/cryst12030425

[B40] Sajjad M. Singh N. Schwingenschlögl U. (2018). Strongly Bound Excitons in Monolayer PtS2 and PtSe2. Appl. Phys. Lett. 112, 043101. 10.1063/1.5010881

[B41] Sanville E. Kenny S. D. Smith R. Henkelman G. (2007). Improved Grid-Based Algorithm for Bader Charge Allocation. J. Comput. Chem. 28, 899–908. 10.1002/jcc.20575 17238168

[B42] Shao C. Ren K. Huang Z. Yang J. Cui Z. (2022). Two-Dimensional PtS2/MoTe2 van der Waals Heterostructure: An Efficient Potential Photocatalyst for Water Splitting. Front. Chem. 10, 847319. 10.3389/fchem.2022.847319 35237564 PMC8882685

[B43] Shen Z. Ren K. Zheng R. Huang Z. Cui Z. Zheng Z. (2022). The Thermal and Electronic Properties of the Lateral Janus MoSSe/WSSe Heterostructure. Front. Mat. 9, 838648. 10.3389/fmats.2022.838648

[B44] Shi D. Wang G. Li C. Shen X. Nie Q. (2017). Preparation and Thermoelectric Properties of MoTe 2 Thin Films by Magnetron Co-sputtering. Vacuum 138, 101–104. 10.1016/j.vacuum.2017.01.030

[B45] Sun M. Chou J.-P. Ren Q. Zhao Y. Yu J. Tang W. (2017). Tunable Schottky barrier in van der Waals heterostructures of graphene and g-GaN. Appl. Phys. Lett. 110, 173105. 10.1063/1.4982690

[B46] Sun M. Chou J.-P. Yu J. Tang W. (2017). Effects of Structural Imperfection on the Electronic Properties of graphene/WSe2 Heterostructures. J. Mat. Chem. C 5, 10383–10390. 10.1039/c7tc03131a

[B47] Sun M. Luo Y. Yan Y. Schwingenschlögl U. (2021). Ultrahigh Carrier Mobility in the Two-Dimensional Semiconductors B8Si4, B8Ge4, and B8Sn4. Chem. Mat. 33, 6475–6483. 10.1021/acs.chemmater.1c01824

[B48] Sun M. Schwingenschlögl U. (2020). δ-CS: A Direct-Band-Gap Semiconductor Combining Auxeticity, Ferroelasticity, and Potential for High-Efficiency Solar Cells. Phys. Rev. Appl. 14, 044015. 10.1103/physrevapplied.14.044015

[B49] Sun M. Schwingenschlögl U. (2021). Structure Prototype Outperforming MXenes in Stability and Performance in Metal‐Ion Batteries: A High Throughput Study. Adv. Energy Mat. 11, 2003633. 10.1002/aenm.202003633

[B50] Sun M. Yan Y. Schwingenschlögl U. (2020). Beryllene: A Promising Anode Material for Na- and K-Ion Batteries with Ultrafast Charge/Discharge and High Specific Capacity. J. Phys. Chem. Lett. 11, 9051–9056. 10.1021/acs.jpclett.0c02426 33044084

[B51] Wang G.-Z. Chang J.-L. Tang W. Xie W. Ang Y. S. (2022b). 2D Materials and Heterostructures for Photocatalytic Water-Splitting: A Theoretical Perspective. J. Phys. Phys. D. Appl. Phys. 55, 293002.

[B52] Wang G. Chang J. Tang W. Xie W. Ang Y. S. (2022a). 2D Materials and Heterostructures for Photocatalytic Water-Splitting: a Theoretical Perspective. J. Phys. D. Appl. Phys. 55, 293002. 10.1088/1361-6463/ac5771

[B53] Wang G. Gong L. Li Z. Wang B. Zhang W. Yuan B. (2020). A Two-Dimensional CdO/CdS Heterostructure Used for Visible Light Photocatalysis. Phys. Chem. Chem. Phys. 22, 9587–9592. 10.1039/d0cp00876a 32322864

[B54] Wang G. Zhang L. Li Y. Zhao W. Kuang A. Li Y. (2020). Biaxial Strain Tunable Photocatalytic Properties of 2D ZnO/GeC Heterostructure. J. Phys. D. Appl. Phys. 53, 015104. 10.1088/1361-6463/ab440e

[B55] Wang G. Zhi Y. Bo M. Xiao S. Li Y. Zhao W. (2020). 2D Hexagonal Boron Nitride/Cadmium Sulfide Heterostructure as a Promising Water‐Splitting Photocatalyst. Phys. Status Solidi B 257, 1900431. 10.1002/pssb.201900431

[B56] Wang S. Ren C. Tian H. Yu J. Sun M. (2018). MoS2/ZnO van der Waals heterostructure as a high-efficiency water splitting photocatalyst: a first-principles study. Phys. Chem. Chem. Phys. 20, 13394–13399. 10.1039/c8cp00808f 29721569

[B57] Wang S. Tian H. Ren C. Yu J. Sun M. (2018). Electronic and Optical Properties of Heterostructures Based on Transition Metal Dichalcogenides and Graphene-like Zinc Oxide. Sci. Rep. 8, 12009. 10.1038/s41598-018-30614-3 30104708 PMC6089903

[B58] Wang S. Ukhtary M. S. Saito R. (2020). Strain Effect on Circularly Polarized Electroluminescence in Transition Metal Dichalcogenides. Phys. Rev. Res. 2, 033340. 10.1103/physrevresearch.2.033340

[B59] Wickramaratne D. Zahid F. Lake R. K. (2014). Electronic and Thermoelectric Properties of Few-Layer Transition Metal Dichalcogenides. J. Chem. Phys. 140, 124710. 10.1063/1.4869142 24697473

[B60] You B. Wang X. Zheng Z. Mi W. (2016). Black phosphorene/monolayer transition-metal dichalcogenides as two dimensional van der Waals heterostructures: a first-principles study. Phys. Chem. Chem. Phys. 18, 7381–7388. 10.1039/c5cp07585h 26899350

[B61] Zhang H. Chhowalla M. Liu Z. (2018). 2D Nanomaterials: Graphene and Transition Metal Dichalcogenides. Chem. Soc. Rev. 47, 3015–3017. 10.1039/c8cs90048e 29700540

[B62] Zhang Q. Ren K. Zheng R. Huang Z. An Z. Cui Z. (2022). First-Principles Calculations of Two-Dimensional CdO/HfS2 Van der Waals Heterostructure: Direct Z-Scheme Photocatalytic Water Splitting. Front. Chem. 10, 879402. 10.3389/fchem.2022.879402 35464209 PMC9021922

[B63] Zhao D. Xie S. Wang Y. Zhu H. Chen L. Sun Q. (2019). Synthesis of Large-Scale Few-Layer PtS2 Films by Chemical Vapor Deposition. AIP Adv. 9, 025225. 10.1063/1.5086447

[B64] Zheng R. Ren K. Yu J. Zhu Z. Sun Q. (2021). Type-II Heterostructure Based on two-Dimensional Arsenene and PtS_2_ With Novel Light Absorption Performance. Third International Conference on Optoelectronic Science and Materials (ICOSM 2021), Hefei, China, December 9, 2021 (SPIE), 182–186.

