# Supplementary material for: The First-Principles Study of External Strain Tuning the Electronic and Optical Properties of the 2D MoTe2/PtS2 van der Waals Heterostructure
Source: Front Chem. 2022 Jul 25;10:934048. doi: 10.3389/fchem.2022.934048 (PMC9357909; doi:10.3389/fchem.2022.934048)
Supplement: Supplementary file 1 [file DataSheet1.docx]

**The First-Principles Study of External Strain Tuning the Electronic and Optical Properties of the 2D MoTe_2_/PtS_2_ van der Waals Heterostructure**

Li Zhang^1^, Kai Ren^2,6^, Haiyan Cheng^3^, Zhen Cui^4^ and Jianping Li^5,^*

^1^Department of Application & Engineering, Zhejiang Institute of Economics and Trade, Hangzhou 310018, China

^2^School of Mechanical and Electronic Engineering, Nanjing Forestry University, Nanjing 211189, China

^3^School of Foreign Languages, Zhejiang University of Finance & Economics Dongfang College, Hangzhou 310018, China

^4^School of Automation and Information Engineering, Xi'an University of Technology, Xi'an, Shaanxi 710048, China

^5^School of Automotive & Transportation Engineering, Shenzhen Polytechnic, Shenzhen, Guangdong 518055, China

^6^School of Mechanical Engineering, Wanjiang University of Technology, Maanshan 243031, China





**FIGURE S1.** The band structure of the MP heterostructure with and without SOC effect by the PBE calculations.


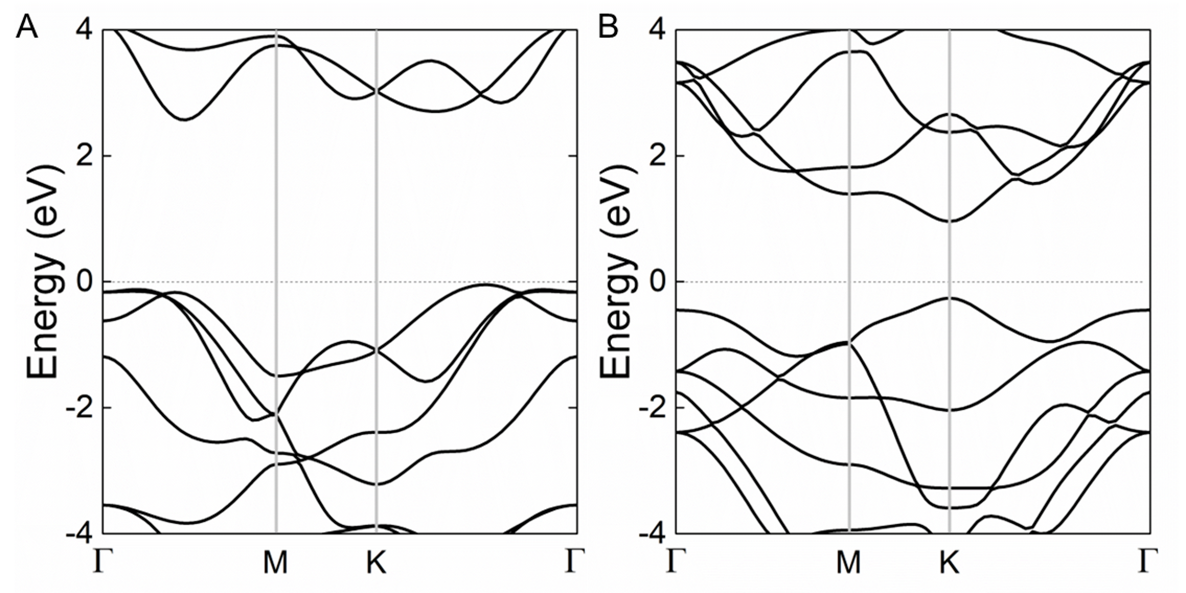


**FIGURE S2.** The band structure of the (A) MoTe_2_ and (B) PtS_2_ monolayers by HSE06 calculations.


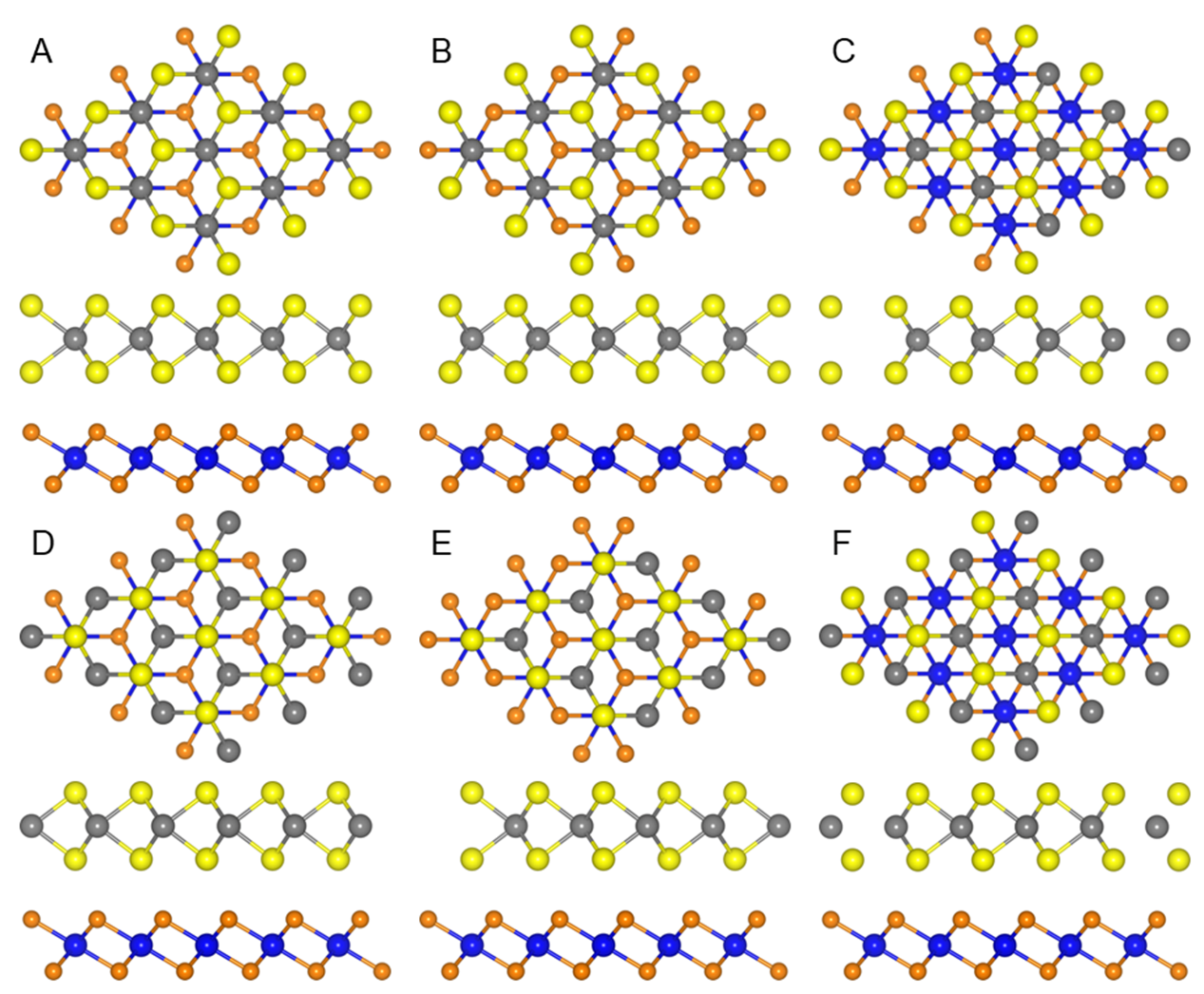


**FIGURE S3.** The stacking styles of the MP vdW heterostructure constructed by (A) MP-1, (B) MP-2, (C) MP-3, (D) MP-4, (E) MP-5, and (F) MP-6, respectively.
